# Interventions for cancer-related pain: Protocol of an umbrella systematic review and network meta-analysis

**DOI:** 10.1097/MD.0000000000017844

**Published:** 2019-11-11

**Authors:** Tao Xu, Hanzhou Lei, Yutong Zhang, Siying Huang, Ziwen Wang, Siyuan Zhou, Jiao Yang, Qianhua Zheng, Jiao Chen, Ling Zhao

**Affiliations:** College of Acupuncture and Moxibustion and Tuina, Chengdu University of Traditional Chinese Medicine, China.

**Keywords:** CRP, network meta-analysis, systematic review

## Abstract

**Background:**

Several treatments are beneficial for patients with cancer-related pain (CRP), and there are numbers of systematic reviews evaluating the effectiveness and safety of these treatments. However, the overall quality of the evidence has not been quantitatively assessed. The aim of this study is to overcome the inconclusive evidence about the interventions of CRP.

**Methods:**

We will perform an umbrella systematic review to identify eligible randomised controlled trials (RCTs). A comprehensive literature search will be conducted in MEDLINE, EMBASE, and the Cochrane library for systematic reviews, meta-analyses and RCTs. We will describe the general information of the RCTs for participants, interventions, outcome measurements, comparisons, and results. Network meta-analysis will be developed to determine the comparative effectiveness of the treatments.

**Results:**

The result of this network meta-analysis will provide direct and indirect evidence of treatments for CRP.

**Conclusion:**

The conclusion of our study will help clinicians and CRP patients to choose suitable treatment options.

**Ethics and dissemination:**

Formal ethical approval is not required, as the data are not individualized. The findings of this systematic review will be disseminated in a peer-reviewed publication and/or presented at relevant conferences.

**PROSPERO registration number:**

CRD42019131721.

## 1 Introduction

Cancer burden rises to 18.1 million new cases and 9.6 million cancer deaths in 2018.^[1]^ More than 70% of the patients reported cancer-related pain (CRP) while undergoing anticancer treatments or during the metastatic, advanced and terminal stages of the disease.^[2,3]^ CRP is generally caused directly by the tumour itself such as compressing on the nerve or inflammation of the organs.^[4]^ It is one of the most common and troublesome symptoms among patients with cancer, which seriously affect the treatment outcomes and quality of life, also imposes a substantial economic burden on healthcare services.^[2]^ Pain management of CRP is a pressing clinical challenge for clinicians.

According to current National Comprehensive Cancer Network (NCCN) guidelines, opioids are recommended as the first-line approach for moderate to severe cancer pain management of CRP; other analgesics include nonsteroidal anti-inflammatory drugs, acetaminophen and antidepressants are also being prescribed.^[2]^ Previous evidence have shown benefits of complementary alternative therapy like acupuncture for supportive in CRP.^[5]^ A network meta-analysis of 81 RCTs compared the effectiveness of various therapeutic classes and individual treatments on chronic cancer pain, found that nonopioid analgesics, NSAIDs, and opioids were the most effective classes, but the lack of non-pharmaceutical therapy limit the strength of these finding.^[6]^ Despite the availability of opioids and other analgesics, side effects and undertreatment are common.^[7]^ A review suggest that undertreatment of chronic cancer pain remains significantly high at 31.8% by practicing oncologist.^[8]^

An important clinical question was raised: which intervention is the best choice for CRP? Little published evidence has compared the efficacy and safety of these interventions in patients with CRP. To address this question, Bayesian network meta-analysis enables a comprehensive analysis through integrating all direct and indirect evidence to compare various interventions. Therefore, we will conduct an umbrella systematic review and network meta-analysis to comprehensively evaluate the effectiveness and safety of various interventions for CRP.

## 2 Methods and analysis

### 2.1 Study design

Considering the large number of interventions for CRP and the availability of numerous systematic reviews that examined the efficacy and safety of these interventions, we will conduct an umbrella systematic review to identify eligible RCTs and a network meta-analysis to determine the comparative effectiveness of the treatments. This protocol report is based on the Preferred Reporting Items for Systematic Reviews and Meta-Analyses Protocols (PRISMA-P) guidelines and has been registered in PROSPERO (CRD42019131721).^[9]^

### 2.2 Search strategy

We will search the electronic databases MEDLINE, EMBASE, and the Cochrane library for systematic reviews from inception to date, which examine the effectiveness of interventions for CRP. These databases will be screened to search for eligible RCTs published subsequently to the date the latest systematic review was conducted. If multiple systematic reviews on the same intervention are found, we will select the latest one. The interventions that we intend to include will be any systemic pharmaceutical intervention and/or combination thereof (including oral, intravenous, transdermal, and subcutaneous routes) for CRP. The search strategy is provided in Table 1. No language restrictions will be used.

**Table 1:**
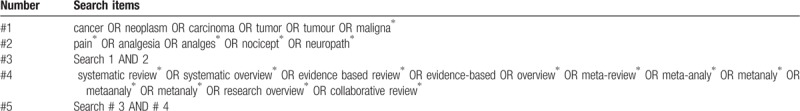
Search strategy for the PubMed database.

### 2.3 Study selection

Two independent reviewers (TX and HZL) will independently identify the titles and abstracts using the search strategy. The inclusion criteria of study will be:

(1) patients have a positive cancer diagnosis;
(2) RCTs with or without blinding,
(3) number of randomisation participants greater than 20 (n ≥ 20);
(4) any systemic pharmaceutical intervention and/or combination thereof.

Non-RCTs, case studies, clinical observations, nonhuman studies, abstracts and unpublished data were excluded from consideration. If there are disagreements between the 2 reviewers, all the authors will discuss and solve the problems.

### 2.4 Data extraction

Two reviewers (TX and HZL) independently extracted the data from the studies, and any disagreements were resolved by discussion and solve with all the authors. Reviewers will extract data using a standard form including information about the country, type of cancer, number of arms, number of participants, drug and dosing regimen, pain scale, study design (placebo or active control), allocation ratio, study duration, and follow-up, characteristics patients (age, sex, duration, and a subclassification of pain types), analgesic outcome measures and measurement time points, results, withdrawals, and adverse events.

### 2.5 Outcome assessment

We planned to include trials measuring pain intensity assessed using validated tools such as Visual Analogue Scale (VAS), Numerical Rating Scale (NRS), Verbal Rating Scale (VRS), Faces Pain Scale - Revised (FPS-R), McGill Pain Questionnaire (MPQ), Colour Analogue Scale (CAS), or any other validated rating scales, which could be linearly transformed to a standardized 100-point scale. The prespecified primary outcome was overall response. The prespecified secondary outcomes were quality of life (defined by validated scales) and any adverse events.

### 2.6 Risk of bias assessment

The methodological quality of RCTs will be evaluated using the Cochrane Collaboration's risk of bias tool. The risk of bias tool consists of 8 domains: random sequence generation; allocation concealment; blinding of participants; blinding of the evaluator; blinding of the outcome assessment; incomplete data; missing data and other. The quality of the body of the evidence related to each of the key outcomes will be assessed using the Grading of Recommendations Assessment, Development and Evaluation (GRADE). This tool provides a rating of ‘high’, ‘moderate’, ‘low’, or ‘very low’ quality, and will provide a ‘weak’ or ‘strong’ recommendation. If there are disagreements between the two reviewers, all the authors will discuss and solve the problems.

### 2.7 Statistical analysis

The effect size of the continuous data will be calculated using the standardised mean difference (SMD). The effect size of the dichotomous data will be calculated using the relative ratio (RR). The RR and the 95% Confidence interval (CI) of each intervention will be calculated and pooled using the random-effect model. The network meta-analysis will conduct with a Bayesian hierarchical random effects model using WinBUGS (version 1.4.3; MRC Biostatistics Unit, Cambridge, United Kingdom) to combine direct and indirect evidence of interventions for CRP.^[10]^

### 2.8 Dealing with missing data

Missing data will be estimated from the published data. We will first contact the correspondence authors to ask for the missing data by email or phone calls. Intention-to-treat (ITT) analysis will be used when available.

### 2.9 Subgroup analysis

In case of possible important heterogeneity, we explored the possible sources using subgroup and meta-regression analyses. Factors such as interventions, control group, baseline measures, sample size, age and sex will be conducted.

### 2.10 Sensitivity analysis

Sensitivity analyses will be performed to verify the robustness of the review conclusions. The impacts of study design, methodological quality, and missing data will be evaluated. Sensitivity analyses were planned by studies considered being at low risk of bias.

## 3 Discussion

This study is expected to provide a ranking of the interventions for CRP, based on comparative effectiveness evidence. We hope that the result would help the physicians and cancer patients to choose their best preferences, after comprehensive consideration of effect estimates, side effects and the costs. We believe that our study will provide important information for health policy makers.

## Author contributions

TX and HZL contributed equally to this manuscript and joint first authors. LZ obtained funding. YTZ, YY, SYH and XL drafted the protocol. The search strategy was developed and will be conducted by TX and SYZ. JC and ZWW will obtain copies of the studies and SYH and YTZ will select the studies to be included. TX, HZL, and JC will extract data from the studies. YY and XL will enter data into RevMan. TX, HZL, and LZ will conduct the analyses. TX, SYZ, YTZ, and YY will interpret the analyses. TX, SYZ, YTZ, and XL will draft the final review and JC and LZ will update the review. LZ will act as an arbiter in the study selection stage. All authors have read and approved the final manuscript.
